# The investigation of the efficiency of basic life support education among high school students: Protocol, design and implementation of an interventional, prospective longitudinal, individually randomised, parallel 1:1 grouped trial

**DOI:** 10.1016/j.resplu.2024.100585

**Published:** 2024-02-28

**Authors:** Csaba Maár, Endre Zima, Bettina Nagy, Ádám Pál-Jakab, Petra Szvath, Boldizsár Kiss, Gábor Fritúz, János Gál, Béla Merkely, Enikő Kovács

**Affiliations:** a Department of Anesthesiology and Intensive Therapy, Semmelweis University, Üllői út 78, Budapest 1082, Hungary; b Department of Anesthesiology and Perioperative Care, Semmelweis University, Üllői út 78, 1082 Budapest, Hungary; c Hungarian Resuscitation Council, Bem rakpart 28, 1011 Budapest, Hungary; d Heart and Vascular Centre, Semmelweis University, Gaál József út 9-11, 1122 Budapest, Hungary; e Health Services Management Training Centre, Semmelweis University, Kútvölgyi út 2, 1125 Budapest, Hungary

**Keywords:** Cardiopulmonary resuscitation, Basic Life Support, Premedical Education Study Skills, Cardiac arrest, Research, Methodology

## Abstract

**Background:**

Basic life support (BLS) skills are crucial not only for healthcare workers but for all lay people as well. Timely recognition of out-of-hospital cardiac arrest (OHCA) and the initiation of BLS by bystanders before the arrival of healthcare personnel may improve survival. There are several methods of spreading BLS skills and improve BLS skill retention among lay people. One of these methods can be the education of adolescent school children. The introduction of mandatory BLS education in schools was very effective in some European countries to increase the rate of bystander BLS.

**Methods/design:**

The current study aims to investigate the efficacy of a BLS training and BLS curriculum among high school children in Hungary. Moreover, the investigators would like to optimise factors influencing skill retention in this first responder group and aim to compare two types of teaching methods: feedback given by the instructor or software-based feedback on the efficacy of chest compressions during the course. This study will be an interventional, assessor blinded, individually randomised parallel group trial recruiting 360 students. BLS skill retention will be assessed at the end of the course, two months after the training and six months after training.

**Discussion:**

The current study will increase our knowledge on the methods educating BLS among high school children. The results will help us to create an effective BLS curriculum at schools.

**Trial registration:** ClinicalTrials.gov: NCT06016153. Prospectively registered on 08/2023.

## Introduction

Sudden cardiac death is one of the most common causes of death in Europe.^1^ Despite the great efforts we have made in the past decades to increase the effectiveness of resuscitation survival rate is still poor. Average, 350,000–700,000 people suffer from out-of-hospital cardiac arrest (OHCA) in Europe, from which resuscitation takes place approximately in 100,000 cases and spontaneous return of circulation could be achieved only in 40,000 cases. Only 3% of all patients who suffered OHCA survive permanently without any impairment of quality of life (Cerebral Performance Category 1).^2^, ^3^

The elements of the survival chain (early recognition of cardiac arrest and immediate call for help, chest compression started as early as possible, early defibrillation (if appropriate), and adequate post resuscitation care) determine the good outcome of cardiopulmonary resuscitation. The chain is as strong as its weakest link; therefore, it is mandatory to enforce all the links that is based on well-established educational and training methods.^4^

The first two links of the chain define the effectiveness of basic life support (BLS), consequently the high quality BLS is the first and very important key that improves the outcome of OHCA patients. However, BLS provider skills deteriorate three to twelve months after learning the BLS procedure if it has not had been applied or practiced during this period of time.^5^, ^6^ Several tools and methods have been investigated previously to improve BLS skill retention. One of them is the usage of systems and tools that reflect the effectiveness of the elements of BLS applied during the process.^7^, ^8^ The application of feedback devices is also beneficial giving an immediate response on the quality of BLS to the provider, so inappropriate action and technical elements, such as the depth and frequency of chest compressions, can be revised and corrected continuously and the chance of return of spontaneous circulation can be improved. However, we would like to point out that the positive effect of feedback devices on survival rates has not yet been proven.^9^ On the other hand, several other educational methods have been also investigated to improve BLS skill retention. It has been proven that taking exam at the end of the BLS practice is more effective than if the students spend the same amount of time with practicing. Nevertheless, it is not clear whether the timing of the exam, namely the duration between the practice and the exam affects skill retention.^10^

In addition, the early education of children and adolescents for BLS is getting highly emphasized in the recent scientific position statements from the European Resuscitation Council (ERC) and International Liaison Committee on Resuscitation (ILCOR).^11^ Young people are important target for BLS education based on its multiple effects. Beside that they can act as multipliers and transmitters of the specific information concerning BLS to their environment, all groups of society could be reached via them. For the distribution of early age provider BLS, “Kids Save Lives” program was launched by the ERC in 2015. Providing resuscitation training in schools (from the age of 12, two hours per year) is one of the most important steps in increasing the rate of bystanders and improving the worldwide survival.^12^, ^13^

We must highlight that the result of an effective BLS education should be the ability to provide high-quality chest compression.

### Aims and objectives

The current study aims to investigate the efficacy of a BLS training and BLS curriculum among 16–19 year old high school adolescence in Hungary. By investigation of the factors influencing short- and long-term skill retention in this age group we aim to compare two types of teaching methods: the feedback concerning the efficacy and quality of chest compression is given by an experienced instructor or by a real-time software during the BLS training. The trial also wants to determine whether the motivation of the students affects BLS skill retention or not.

## Methods

### Design

The trial is an anonymous, interventional, prospective longitudinal, assessor blinded, individually randomised, parallel 1:1 grouped trial to determine and compare the efficiency of the two teaching methods: feedback given by the instructor or by a software and CPR-sensor module on the efficacy and quality of chest compressions during the course. This protocol has been written in concordance with the SPIRIT guidelines (Table 1 and Appendix 1).^14^

**Table 1:** Trial registration data set (BLS: Basic Life Support).

Data category	Information
Registry	ClinicalTrials.gov ID: NCT06016153
Date of registration	08/2023
Human Subjects Review	Board Status: Approved Approval Number: 160/2023 Board Name: Semmelweis University Regional and Institutional Committee of Science and Research Ethics Board Affiliation: Semmelweis University Email: titkarsag.kutatasetikai-bizottsag@semmelweis-univ.hu Address: 1091 Budapest, Üllői út 93.
Primary sponsor	Semmelweis University This funding source had no role in the design of this study and will not have any role during its execution, analyses, interpretation of the data, or decision to submit results.
Secondary sponsor	none
Central Contact Person	Uzonka Szabolcsi szabolcsiu@gmail.com
Central Contact Backup	Enikő Kovács PhD kovacs.eniko2@med.semmelweis-univ.hu
Study Officials	Endre Zima PhD Study Principal Investigator, Semmelweis University
Public Title	The Efficacy of Basic Life Support Education Among Teenagers
Scientific Title	The Investigation of the Efficacy of Basic Life Support Education Among High School Students
Countries of recruitment	Hungary
Health condition(s) or problem(s) studied	The quality of Basic Life Support
Intervetion(s)	BLS training, feedback given by the instructor or a CPR analyser software
Key inclusion criteria	• High school teenagers (aged 16–19 years) participating in the education of Óbudai High School, Budapest • Written informed consent received from participants and their parents to participate in the study
Key exclusion criteria	• Missing written informed consent provided by the student or parent • Any injury or health issue influencing the efficacy of BLS skill
Study type	• Study Type: Interventional • Interventional Study Model: Parallel Assignment • Number of Arms: 2 • Masking: Single (Outcomes Assessor) • Allocation: Randomized
Date of first enrolment	December 2023
Target sample size	360
Recruitment status	Recruiting
Primary outcome(s)	Results of the assessment at the end of the training
Key secondary outcome(s)	Results of the assessments two and six months after the training

In addition, a questionnaire will be used to collect data on students’ characteristics and motivation (see Appendix 2).

The Semmelweis University Regional and Institutional Committee of Science and Research Ethics approved our study (Approval number 160/2023). In the case of any protocol modifications, the Board will be notified by author EZ via email and official letter.

### Setting, criteria

#### Participants

The research focuses on students aged between 16 and 19 years from a secondary school in Budapest (Óbudai High School). We plan a pilot study from the results of the first 60 schoolchildren to be able to make a power analysis and an effect size.

The main trial is planned to involve 330–360 students. If necessary, we will consider to involve more high schools to participate.

A written informed consent provided from the participants and their parents is needed to participate in the study before any trial procedures occur (see Appendix 3 for a sample of the written informed consent form). The text of the consent will be sent out before the study to participants and the written formats will be collected by instructors on-site, at the beginning of trainings. In addition, students will need to fill out a brief questionnaire about their basic characteristics and future educational motivation (gender, age, prior participation in BLS course, plans about future specialisation, etc). The participation in the study will be voluntary. Students can indicate any time their withdrawal from the trial.

Student eligibility for the trial must comply with all the criteria listed and reviewed in Table 2.

**Table 2:** Inclusion and exclusion criteria (BLS: Basic Life Support).

*Inclusion criteria*	*Exclusion criteria*
High school teenagers participating in the education of Óbudai High School, Budapest	No written informed consent provided by the student and/or parent. Unwilling to participate on site
Written informed consent received from participants and their parents to participate in the study	Any injury or health issue influencing the efficacy of BLS performance

#### Randomisation

**Fig. 1:**
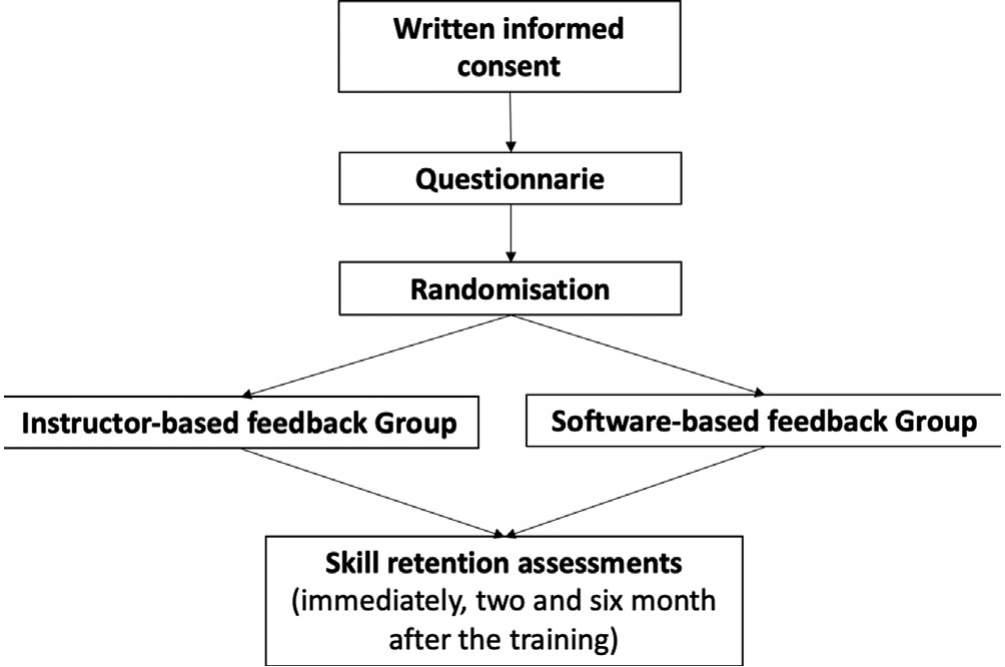
Trial flow diagram.

Students will be simply randomised in two groups by randomly dividing the groups. Participants randomised to **Instructor Group** will take part in a 90-minute-long BLS training and get a feedback based on the own experience of the instructor. Students randomised to **Software Group** will also take part in a 90-minute-long BLS training, but they will receive feedback by a real-time software-based feedback system (Ambu Man model C Manikin, Ambu A/S, Ballerup, Danish Kingdom, and InnoMed CardioAid-1 Trainer AED, Innomed Inc., Budapest, Hungary). There will be four BLS training occasions for the 360 students organized during school time with the help of teachers. Students applying for the first two occasions of the trainings will be randomised to Instructor Group and students applying to the last two occasions will be randomised to Software Group. Two authors (CsM and EZ) will be responsible for the randomisation and students’ enrolment to the study. All students will receive a code that will be added to a Microsoft Excel spreadsheet (Microsoft Corporation, Redmond, WA) with the name and grouping information of the students. To secure students’ anonymity, only the codes will be used during the evaluation and data process. Due to the nature of the study and intervention students and instructors will know which group they belong to during the trainings. Skill retention assessment with an observational checklist will be performed at the end of the training, two months later and six months later by our instructors to evaluate short- and long-term BLS skill retention. All data regarding the students’ performance will be added to another Microsoft Excel spreadsheet. The instructors will be blinded during skill retention assessment and will not be aware of student grouping. The assessor instructors will be independent from the instructors educating during the trainings. Fig. 1 summarizes the flow of the trial.

### Trial interventions

#### Instructors

Instructors teaching CPR at our department either hold an ERC Certificate or are trained based on the ERC Educational Guidelines and Principles.^6^

In order to make BLS trainings and data recording unified, the Instructors participated on an internal pre-training standardised and tailored to the high school BLS education.

The teacher to student ratio will be 1:6 and in some exceptional cases no more than 1:7 during BLS practices.

#### BLS training

Students will receive a 90-minute long BLS training according to the actual ERC Guideline. Peyton’s Four-Step Approach will be the teaching method that will be used during BLS training. It contains four important steps: 1. real-time instructor demonstration of BLS on a manikin without any explanations; 2. repeated instructor demonstration with detailed explanation followed by students’ questions if any; 3. instructor demonstration based on a volunteer or selected student’s instructions; 4. student’s demonstration with instructor’s help if needed.^6^

After the four-step demonstration students will work in pairs to practice BLS. They will have to solve simple scenarios and have enough time to practice the whole algorithm. All participants in the **Instructor Group** will receive feedback separately based on the instructor’s own experience by following the key points of the checklist. Beside the presence of important steps in BLS, the instructor will observe and give feedback based on experience by strengthening the key points of the checklist (see Appendix 4). A software-based real-time system will be used in **Software Group**. It will give feedback about the qualities of the chest compression (depth, frequency, chest release, rhythm, etc.) and ventilation. In addition, the instructor will give feedback regarding the presence of the most important steps of BLS.

The key messages during the training will be the proper initiation of chain of survival by checking environmental safety, checking the victim’s response to voice and tactile stimuli, opening the airways, checking the breathing, asking for help and performing high-quality chest compressions combined with rescue breathing in 30:2 ratio during BLS.^15^ The components of high quality chest compressions and rescue breaths are detailed in Appendix 4.

### Outcome, data collection, analysis

#### Skill retention assessment

We will perform skill retention assessments immediately, then two and six months after the BLS training to evaluate the short- and long-term efficacy of the two methods of the BLS training. A standardized checklist will be used containing all the crucial order and elements of the BLS procedure (see Appendix 4). We will use the same assessment method for the Instructor and Software groups.

Students will need to solve the same scenarios during the practices and skill retention assessment. They will work in pairs, but only the primary responder will be evaluated. The length of a scenario will be two minutes long and the following steps of BLS will be evaluated by two independent instructors who had not thought the students during their BLS practice: 1. controlling the safety of environment; 2. examining consciousness; 3. opening the airways; 4. testing the vital signs; 5. calling for help/ for advanced life support team or for AED; 6. performing high- quality chest compressions (right position of the hands on the chest, rate of chest compressions between 100–120 per minute, depth of chest compressions between 5 and 6 cm, release and recoil of the chest, duty cycle, 30 compressions); 7. rescue ventilation (opening the airways, 2 effective breaths with a time less than 10 seconds); 8. maintaining the 30:2 compression to ventilation ratio.

Instructors will use a checklist indicating if a step was correct or not and mark the exact rate and depth of chest compressions measured with the software of Innomed CardioAid-1 Trainer AED. A step will be considered correct if it was performed by the ERC Guidelines in at least 75% of the time during the assessment (cut off: 0.75). The eight steps will be evaluated separately. If a step was correct, the student will receive one point (incorrect steps will be evaluated with zero) and a summary score (range from 0 to 15) will also be calculated by adding up the individual BLS Skill scores.

#### Primary outcome

BLS skill scores will be measured immediately at the end of the training based on a checklist reviewed above (see Appendix 4). The assessment will be performed by one or two instructors and the Innomed CardioAid-1 Trainer AED system.

#### Secondary outcome

BLS skill scores will be measured two and six months after the educational session, based on the procedure reviewed above. Skill retention assessment occasions will be organized in the school with the help of teachers in the same way as the training, during school time.

The influence of professional motivation (planning a profession in health care or not) on BLS performance will also be analysed.

#### CPR skills assessment and data collection

Students’ characteristics and skill retention assessments results (BLS Skill Scores) will be collected in a Microsoft Excel spreadsheet and compared between the Instructor Group and Software Group at each time points of the study, respectively. For the next step, the overall results of skill retention assessments will be evaluated and the results will be compared to assess the efficacy of the BLS education method. One of the authors (ÁPJ) will check the quality of data and will store them. Due to the nature of the study (manikin-based study investigating education methodology) data monitoring committee will not be set up. Only the authors will have access to the data. The participants can check their own results and have access to them for request. Results will be communicated via scientific publication with anonymised data. Moreover, public media presentations are planned with the written consent from participants and their parents.

#### Statistical analysis

Skill retention assessment results, basic characteristics, and data regarding future professional plans with consequential motivation will be analysed. Categorical variables will be described as numbers and percentages, while continuous variables will be described as mean and standard deviation. Chi-square test will be applied for categorical variables and t-test or Mann-Whitney test (for the comparison of Instructor Group and Software groups) will be used for continuous variables. The overall results of a group of the three skill retention assessments will be compared with Friedmann-test. The influence of motivation on BLS performance will be evaluated with logistic regression.

If only 5% of data (or less) will be missing, they will be simply left out from the analysis. If more than 5% of data will be missing, multiple imputation will be performed. The level of significance will be set at p < 0.05. SPSS Statistics 22.0 (Armonk, New York, US) will be used for statistical analysis.

## Discussion

All of the elements in the chain of survival in OHCA are crucial to gain sufficient outcome. It is already well-known that BLS skills deteriorate in three to twelve months if not practiced or retrained regularly.^16^, ^17^ Improving BLS skill retention among lay people and healthcare professionals is one of the most important tasks aiming the improvement of cardiopulmonary resuscitation efficacy to increase survival rates after cardiac arrest. There are several methods to maintain or prolong skill retention.^18^, ^19^ The actual ERC Guidelines on Education recommend learner adapted programs, technology enhanced learning, and annual short competency refreshers.^6^

Another effective way to improve the rate of survival in OHCA worldwide is training as many lay people to provide CPR as bystanders or first responders. An easy and long-lasting opportunity to multiply the number of lay bystanders is to reach the population and all groups of society via the education of schoolchildren aged from 12 or even youngers.^20^ CPR education among them could lead to a significant improvement in global health by increasing the awareness of CA and the willingness to provide BLS.^21^ The result is a possible increase in the number of OHCA survivors, nevertheless it has a social benefit with the increasing number of enthusiastic and responsible young people who are trained to provide proper BLS.^22^

After introducing “Kids Save Lives” program, the rate of bystander CPR in Denmark almost doubled after five years. Nevertheless, a threefold improvement was shown in survival following OHCA over ten years.^23^

“Kids Save Lives in Hungary” (KSLH) was introduced in 2018. Thousands of 5–18 years old children were taught in several kindergartens, primary and secondary schools via in-person education programs. Due to the COVID-19 pandemic regulations, in-person programs were transferred to online education. Though KSLH had valuable results, multi-sectoral co-operation would be needed to improve the effectiveness.^24^

There are several teaching methods to educate CPR, but still there is a lack of recommendation or consensus statement about the best feedback systems used during CPR training. A recent observational study has evaluated chest compression quality assessment in Brasil involving 104 schoolchildren aged 11–17 years with no previous CPR training. This prospective longitudinal study compared two methods: instructor-led checklist versus real-time software system. It showed moderate to high agreement percentages between the two evaluation methods in chest compression rate (68.3%), depth (79.8%), and release (91.3%). Only 38.5% of subjects performed high quality chest compressions during CPR training. The authors concluded that the observational checklist method seemed to overestimate consistently the student CPR performance compared to the real-time software. In addition, they found that better performance was associated with age regardless of sex and body mass index.^25^ This study has not investigated the long-term effect of any method and was made on a small group of students.

Our trial investigates the short- and long-term efficacy of our 90-minute long standard BLS training held for young people (16–19 years) including feedback given by the instructor based on his/her own experience and observation and a real-time software-based teaching method.

## Conclusion

Proper skills are crucial to provide high-quality BLS. We can gain outcome by educating lay people. One of the great opportunities to multiply the numbers of BLS providers is the training of adolescents. We will investigate our teaching method in a high school educational setting in our study. The results of this trial will provide important findings and evidence about the efficacy of BLS training technique and may help to reduce OHCA mortality by improving BLS skill retention.

## Funding

This research did not receive any specific grant from funding agencies in the public, commercial, or not-for-profit sectors.

## CRediT authorship contribution statement

**Csaba Maár:** Writing – original draft, Project administration, Investigation, Formal analysis, Data curation. **Endre Zima:** Writing – original draft, Methodology, Data curation, Conceptualization. **Bettina Nagy:** Project administration, Formal analysis. **Ádám Pál-Jakab:** Project administration, Formal analysis. **Petra Szvath:** Project administration, Formal analysis. **Boldizsár Kiss:** Project administration, Formal analysis, Data curation. **Gábor Fritúz:** Methodology, Conceptualization. **János Gál:** Writing – review & editing. **Béla Merkely:** Writing – review & editing. **Enikő Kovács:** Writing – review & editing, Methodology, Investigation, Conceptualization.

## Declaration of competing interest

The authors declare that they have no known competing financial interests or personal relationships that could have appeared to influence the work reported in this paper.

## Appendix A Supplementary material

Supplementary data 1 SPIRIT checklist.

Supplementary data 2 Questionnaire to survey pupil motivation.

Supplementary data 3 Written Informed Consent Form.

Supplementary data 4 Skill Retention Assessment Form.
