# Supplementary material for: The investigation of the efficiency of basic life support education among high school students: Protocol, design and implementation of an interventional, prospective longitudinal, individually randomised, parallel 1:1 grouped trial
Source: Resusc Plus. 2024 Feb 28;18:100585. doi: 10.1016/j.resplu.2024.100585 (PMC10909624; doi:10.1016/j.resplu.2024.100585)
Supplement: Supplementary data 2 — Questionnaire to survey pupil motivation. [file mmc2.docx]

**Appendix 2.** – Questionnaire to collect data on basic characteristics and student motivation (BLS: Basic Life Support; CPR: Cardiopulmonary resuscitation)

| Name: |  |  |  |
| --- | --- | --- | --- |
| Date: |  |  |  |
| Gender: |  |  |  |
|  | male | female | other |
| Year of birth: | |  |  |
| Height: |  | cms |  |
| Weight: |  | kgs |  |
| Have you ever participated in a BLS course? | | | |
|  | yes | no |  |
| Have you ever participated in a CPR? | | |  |
|  | yes | no |  |
| If you participated in a BLS course, how many months ago? | | | |
| If you participated in a BLS Course, what was the purpose? | | | |
|  | Driving license | |  |
|  | Course organised by the European Resuscitation Council/ Hungarian Resuscitation Council | | |
|  | First aid course organised by the Malteser Charity | | |
|  | Sport event or doing sport | | |
|  | Other: |  |  |
| Do you know what would you like to study after high school graduation? | | | |
|  | I don't know |  |  |
|  | I would like to choose a healthcare profession | | |
|  | I don't want to choose a healthcare profession | | |
