# Supplementary material for: The investigation of the efficiency of basic life support education among high school students: Protocol, design and implementation of an interventional, prospective longitudinal, individually randomised, parallel 1:1 grouped trial
Source: Resusc Plus. 2024 Feb 28;18:100585. doi: 10.1016/j.resplu.2024.100585 (PMC10909624; doi:10.1016/j.resplu.2024.100585)
Supplement: Supplementary data 3 — Written Informed Consent Form. [file mmc3.docx]

**Appendix 3.** – Written Informed Consent Form (BLS: Basic Life Support, CPR: Cardiopulmonary Resuscitation)

Dear Parent, dear Student,

Sudden cardiac death is one of the most common causes of death in Hungary today, however, its survival could be significantly improved if it is detected and treated right in time. Therefore, the education of lay people to provide Basic Life Support (BLS) is essential to improve outcome. On behalf of Semmelweis University (Városmajor Heart and Vascular Centre, Department of Anesthesiology and Intensive Therapy) and the Hungarian Resuscitation Council, we have been launching a program involving high school students to be taught the cardiopulmonary resuscitation (CPR) procedure and skills.

Within this program our professionals trained in both healthcare and education will teach practical BLS skills in the school of your child. During this education your child will take part in a 90-minute long simulation training on BLS. By completing it he/she will be able to recognize sudden cardiac death and learn the initial steps of treatment. The location of this course is the school of your child.

In addition, we would like to improve the efficiency of our educational methodology continuously - and concomitantly the efficiency of the treatment of the patients who suffered from circulatory arrest-, which means that as the part of the course we are investigating a scientific point on educational methodology. Regarding this we would like to ask your child to fill out a short questionnaire with basic data and to participate in a short (2 minute) practical skill retention assessment completed immediately, two and six months after the course. During the skills assessment sessions we anonymously register and evaluate the characteristics of your child’s BLS skills.

Our study would like to evaluate efficacy of the BLS education and investigate the short- and long-term skill retention associated with our teaching methods. Practising before the skill assessment is not available due to the protocol of this study.

Only the instructors who participate in this study have the right to access the data on questionnaire and the skill retention test performance and they use them as completely confidential.

I, the undersigned …………….………………… (date of birth: ……….………....….) agree that my child's (child's name: ............................. ) answers to the questionnaire, as well as the BLS performance data provided during course and assessments will be used anonymously for scientific purposes.

I confirm that I had received information about the study before signing this consent form.

I understand the purposes, methods of the investigation and I accept them. I am aware that the data obtained in the research program will only be used for research purposes. I also received the information above in writing.

I agree that from the data obtained during the investigation to be exerted in the scientific processing and publication while keeping my child's personal data confidential. Furthermore, I acknowledge that neither my child nor I will receive financial compensation for voluntary participation in this research program.

Budapest, 2023 __ month __day

_____________________________ ________________________

instructor providing the information child’s and parent's signature (if the child is under 18 yo.)
