# Supplementary material for: The investigation of the efficiency of basic life support education among high school students: Protocol, design and implementation of an interventional, prospective longitudinal, individually randomised, parallel 1:1 grouped trial
Source: Resusc Plus. 2024 Feb 28;18:100585. doi: 10.1016/j.resplu.2024.100585 (PMC10909624; doi:10.1016/j.resplu.2024.100585)
Supplement: Supplementary data 4 — Skill Retention Assessment Form. [file mmc4.docx]

**Appendix 4. –** Skill Retention Assessment Form (ID: identification; BLS: Basic Life Support; AED: Automated External Defibrillation; CC: chest compressions)

ID:

Date:

Group by randomization

Assessment: 1st // 2nd // 3rd

If a step is correct, put one point. If not correct, put a zero.

| **BLS SKILL RETENTION ASSESSMENT FORM** | |
| --- | --- |
| controlling the safety of environment *(look around)* |  |
| examining consciousness *(shake and ask loudly)* |  |
| **opening the airways** |  |
| **checking the breathing/ testing the vital signs** |  |
| **calling for help / for advanced life support team or for AED** |  |
| **doing quality chest compressions (CC)** | |
| **correct position of the hands on the chest** |  |
| **rate of CC between 100-120 per minute** |  |
| **depth of CC between 5-6 cms** |  |
| **release and recoil of the chest** |  |
| **duty cycle** |  |
| **30 CC** |  |
| **rescue ventilations** | |
| **opening the airways** |  |
| 2 effective breaths |  |
| **time spent without CC is less than 10 sec** |  |
| **maintaining the 30:2 compression to ventilation ratio** |  |
| **BLS Skill Score** |  |

Comments:

Examiner(s), signature(s): ................................................
